# Comparative analysis of the gut bacteria and fungi in migratory demoiselle cranes (*Grus virgo*) and common cranes (*Grus grus*) in the Yellow River Wetland, China

**DOI:** 10.3389/fmicb.2024.1341512

**Published:** 2024-03-20

**Authors:** Ziteng Li, Tianfeng Duan, Lin Wang, Jiawei Wu, Yajie Meng, Dulan Bao, Li Gao, Li Liu

**Affiliations:** Faculty of Biological Science and Technology, Baotou Teacher's College, Baotou, China

**Keywords:** gut bacteria, gut fungi, demoiselle crane (*Grus virgo*), common crane (*Grus grus*), Yellow River Wetland

## Abstract

**Introduction:**

Gut microbiota are closely related to the nutrition, immunity, and metabolism of the host and play important roles in maintaining the normal physiological activities of animals. Cranes are important protected avian species in China, and they are sensitive to changes in the ecological environment and are thus good environmental indicators. There have been no reports examining gut fungi or the correlation between bacteria and fungi in wild Demoiselle cranes (*Grus virgo*) and Common cranes (*Grus grus*). Related research can provide a foundation for the protection of rare wild animals.

**Methods:**

16S rRNA and ITS high-throughput sequencing techniques were used to analyze the gut bacterial and fungal diversity of Common and Demoiselle cranes migrating to the Yellow River wetland in Inner Mongolia.

**Results:**

The results revealed that for gut bacteria α diversity, Chao1 index in Demoiselle cranes was remarkably higher than that in Common cranes (411.07 ± 79.54 vs. 294.92 ± 22.38), while other index had no remarkably differences. There was no remarkable difference in fungal diversity. There were marked differences in the gut microbial composition between the two crane species. At the phylum level, the highest abundance of bacteria in the Common crane and Demoiselle crane samples was Firmicutes, accounting for 87.84% and 74.29%, respectively. The highest abundance of fungi in the guts of the Common and Demoiselle cranes was Ascomycota, accounting for 69.42% and 57.63%, respectively. At the genus level, the most abundant bacterial genus in the Common crane sample was *Turicibacter* (38.60%), and the most abundant bacterial genus in the Demoiselle crane sample was *Catelicoccus* (39.18%). The most abundant fungi in the Common crane sample was *Penicillium* (6.97%), and the most abundant fungi in the Demoiselle crane sample was *Saccharomyces* (8.59%). Correlation analysis indicated that there was a significant correlation between gut bacteria and fungi.

**Discussion:**

This study provided a research basis for the protection of cranes. Indeed, a better understanding of the gut microbiota is very important for the conservation and management of wild birds, as it not only helps us to understand their life history and related mechanisms, but also can hinder the spread of pathogenic microorganisms.

## 1 Introduction

The gut microbiota is an important component of animal health. The gut microbiota plays a pivotal role in animal health and aids in essential functions such as digestion, vitamin synthesis, pathogen resistance, and immunity enhancement (Qin et al., 2010; Diaz et al., 2011; Al-Asmakh et al., 2014). This microbiota can even affect host behavior (Grond and Jumpponen, 2018). Due to human interference, climate change, and other factors, the biodiversity on Earth has been decreasing with every passing year. Increased awareness of environmental protection among people has led to a focus on the preservation of biodiversity. Research examining the gut microbiota can aid our understanding of the life history of animals and their related mechanisms that play a very important role in wildlife protection and management. This study aimed to analyze the community composition and differences in gut bacteria and fungi between the common crane and the demoiselle crane during their migration period in the Yellow River Wetland of Inner Mongolia.

There are a total of 15 species of Gruidae birds belonging to the order Gruiformes worldwide. The demoiselle crane (*Grus virgo*) and the common crane (*Grus grus*) are part of the Gruiformes order, the Gruidae family, and the Grainae subfamily. Both these species are listed as national second-level key protected animals in China. Early research examining cranes primarily focused on macro aspects such as their habitat (Li, 1999; Zhao et al., 1999), behavior (Guo, 2005), artificial reproduction (Zou et al., 2005), migration (Gao et al., 2007; Jiao, 2015), and other characteristics. As cranes are sensitive to changes in the ecological environment, they act as good environmental indicators. China possesses the richest crane population (Su et al., 2000). In recent years, the crane population in China has declined sharply driven by the impact of human activities such as environmental pollution, wetland destruction, habitat fragmentation, and the lack of specialized management institutions. So, there is an urgent need to protect their population from further decline. The study of the gut microbiota in cranes can provide an important pathway for the protection of animals.

During the autumn migration period, thousands of demoiselle cranes and common cranes arrive in the wetlands along the Yellow River in Inner Mongolia for energy replenishment. After a brief stop, the demoiselle cranes continue their journey to their wintering area, whereas the common cranes remain in the wetlands throughout winter. To investigate the survival status of the two different crane species during the migration period in the Yellow River Wetland of Inner Mongolia, their gut bacterial and fungal community composition and differences were analyzed using the marker 16S rRNA genes and internal transcribed regions (ITS) by high-throughput sequencing that is widely utilized for the analysis of gut microbiota diversity. This study provides a scientific basis for the formulation of bird protection policies and the protection of cranes in the Yellow River Wetland in Inner Mongolia.

## 2 Material and methods

### 2.1 Ethics statement

This study was performed in accordance with the recommendations for animal care and ethics of China. Non-invasive techniques were used to collect fecal samples. The Animal Ethics and Welfare Committee of Baotou Teachers College approved this study.

### 2.2 Study area

Samples from common cranes were collected in Dalate Banner, Ordos City (109°00′-110°45′ E, 40°00′-40°30′ N), located in Inner Mongolia. A temperate continental monsoon climate prevails in the region. The average annual temperature of the region is 6.58°C and it receives an average annual precipitation of 306.7 mm. This area is a resting place for migratory birds such as swans, ruddy shelducks (*Tadorna ferruginea*), and common cranes. The sampling site for the common cranes was the southern bank of the Yellow River Wetland in Dalat Banner (109°57′E, 40°29′N).

Samples from demoiselle cranes were collected in Darhan Maoming'an United Banner, Baotou city (110°16′-111°25′ E, 41°20′-42°40′ N). It is located on the Inner Mongolian Plateau and a semi-arid continental climate prevails in the region as it lies in the middle temperate zone. The annual average temperature in this region is 4.2°C and it receives an average annual precipitation of 256.2 mm. Xihe Township, Darhan Maoming'an United Banner, an important resting place for the demoiselle cranes, was the sampling site for these animals (110°16′ E, 41°32′ N) in the present study.

### 2.3 Sample collection

Fecal samples were collected from the resting places of the demoiselle and common cranes during the autumn migration period and the wintering period from October to December 2021. Based on previous research, the habitat of a large population of over 200 cranes was selected as the sampling site (Liu et al., 2022b). After the birds left the habitat, a large amount of fresh feces remained. The fresh fecal samples were collected, surface debris was removed, and the samples were stored in paper bags. Disposable gloves were changed while collecting each sample to avoid cross-contamination. To ensure that the samples were obtained from different individuals, the sample collection interval was maintained at >2 m. A total of 11 samples were collected, which included five samples from the demoiselle cranes and six samples from the common cranes. All samples were taken to the laboratory and stored at −80°C for further analysis.

### 2.4 DNA extraction, PCR amplification, and high-throughput sequencing

Total DNA was extracted from each sample using a fecal sample DNA extraction kit (D4015; Omega, Inc., Georgia, USA). The primers 341F (5′-CCTACGGGNGGCGWGCAG-3′) and 805R (5′-GACTACHVGGGTATCTAATCC-3′) were employed to amplify the bacterial 16S rRNA gene V3-V4 hypervariable region (Liu et al., 2022a). The primers ITS1F (5′-ACTTGGTC ATTTAGGAGAAGAGTAA-3′) and ITS2R (5′-GCT GCGTTCTTCATCGATGC-3′) were used to amplify the fungal ITS gene region (Su et al., 2022). Then, 2% agarose gel electrophoresis was used to detect the PCR products, and the PCR products were then purified, quantified, amplified, and sequenced on an Illumina Novaseq 6000 platform (Biomarker Technologies).

### 2.5 Data analysis

Paired-end reads were assigned to samples based on their unique barcodes and truncated by removing the barcode and primer sequences. Paired-end reads were merged using the FLASH software. Raw reads were quality-filtered under specific filtering conditions to obtain high-quality clean reads using fqtrim (v0.94) (Liu et al., 2022a). According to previous studies, 97% sequence identity was utilized to cluster the reads to the operational taxonomic unit (OTU) (Stackebrande and Goebel, 1994) using Usearch software (Edgar, 2013). BLAST searches were used for sequence alignment, and the feature sequences were annotated using the SILVA database for each representative sequence. For measuring the operational taxonomic unit (OTU) level α-diversity of the species, the ACE, Chao1, Simpson, and Shannon indexes were employed to analyze the α-diversity of the gut microbiota in these samples using QIIME2 2020.6 software. Non-metric multidimensional scaling (NMDS) based on unweighted UniFrac distance matrixes was employed to analyze the beta diversity of bacterial communities in different samples using QIIME software. Bray–Curtis NMDS was employed to analyze the β-diversity of fungal communities in different samples using QIIME software, and analysis of similarities (ANOSIM) was used to test if the β-diversity was significantly different using the R language vegan package (*P* < 0.05). For linear discriminant analysis (LDA), the effect size (LEfSe) was employed to analyze bacteria and fungi, and significant differences between the groups (LDA > 4.0, *P* < 0.05) were determined. Briefly, LEfSe analysis with an LDA threshold of >4 was assessed using the non-parametric factorial Kruskal–Wallis sum-rank test, and the (unpaired) Wilcoxon rank-sum test was then used to identify the most differentially abundant taxa. Figures were plotted using R software (Logue et al., 2017) with the “ggplot2” package. Spearman correlation analysis was performed to analyze the correlation between gut bacteria and fungi using R language (*P* < 0.05).

## 3 Results

### 3.1 Sequence statistics and OTU cluster analysis

A 16S rRNA high-throughput sequencing assay was employed to analyze the gut bacteria of common cranes and demoiselle cranes, and the results showed that a total of 880,393 sequences were obtained from six common crane (HH) samples and five demoiselle cranes (SYH) samples. After filtering and screening, 794,192 effective sequences were obtained, and these accounted for 90.21% of the total sequences. A total of 557 OTUs were obtained from these 11 samples, and the number of OTUs obtained from each sample is presented in Table 1. A total of 404 OTUs were acquired from six HH samples and 530 OTUs from five SYH samples. The average OTU number of HH samples was significantly lower than that of SYH (225.67 ± 45.43 vs. 366.80 ± 109.57) (*P* < 0.05).

**Table 1:** Basic information of high-throughput sequencing of 16S rRNA gene of gut bacteria in common cranes and demoiselle cranes.

**Sample**	**Raw reads**	**Clean reads**	**Effective reads**	**Number of OTUs**	**Number of taxa of different taxonomic categories**				
					**Phylum**	**Class**	**Order**	**Family**	**Genus**
HH1	80,152	79,857	79,049	273	15	24	60	101	167
HH2	79,933	79,634	78,922	163	14	18	50	81	120
HH3	79,876	79,590	78,481	202	13	18	53	86	140
HH4	79,854	79,591	78,941	216	14	20	56	93	147
HH5	80,400	80,106	79,335	216	13	19	55	93	144
HH6	79,906	79,596	78,793	284	16	23	64	101	165
SYH1	80,135	79,836	75,158	478	15	21	59	108	222
SYH2	80,352	79,993	78,465	265	14	19	50	88	145
SYH3	79,928	79,498	77,893	340	15	22	58	100	180
SYH4	79,916	79,622	74,910	486	16	22	59	106	227
SYH5	79,941	79,550	76,892	265	15	20	49	79	139

HH, common cranes; SYH, demoiselle cranes.

ITS high-throughput sequencing was employed to analyze the gut fungi of common cranes and demoiselle cranes, and the results showed that a total of 873,678 sequences were obtained from six common crane (HH) samples and five demoiselle crane (SYH) samples. After filtering and screening, 837,647 effective sequences were obtained that accounted for 95.88% of the total sequences. A total of 915 OTUs were obtained from these 11 samples, and the number of OTUs obtained from each sample is presented in Table 2. A total of 812 OTUs were obtained from the six HH samples, and 793 OTUs from the five SYH samples. The average number of OTUs in HH samples was higher than that of SYH, but there were no significant differences (369.50 ± 133.10 vs. 331.20 ± 171.86) (*P* > 0.05).

**Table 2:** Basic information of high-throughput sequencing of ITS gene of gut fungi in common cranes and demoiselle cranes.

**Sample**	**Raw reads**	**Clean reads**	**Effective reads**	**Number of OTUs**	**Number of taxa of different taxonomic categories**				
					**Phylum**	**Class**	**Order**	**Family**	**Genus**
HH1	79,751	77,682	77,169	290	8	25	53	94	150
HH2	73,773	71,354	70,576	477	9	31	64	120	208
HH3	79,898	77,689	76,990	398	10	29	57	108	184
HH4	80,148	77,993	76,317	563	10	35	71	137	244
HH5	8,0131	77,418	76,630	269	8	27	52	94	146
HH6	80,047	78,226	77,956	220	11	28	56	87	129
SYH1	79,890	77,431	75,868	261	8	25	53	92	140
SYH2	80,231	78,008	76,955	638	10	35	75	150	263
SYH3	79,884	77,677	77,210	257	9	25	49	90	141
SYH4	79,869	77,999	77,034	264	8	25	54	87	129
SYH5	80,056	76,401	74,942	236	9	27	52	95	137

HH, common cranes; SYH, demoiselle cranes.

### 3.2 α-diversity analysis of the gut microbiota of the two species of cranes

**Figure 1:**
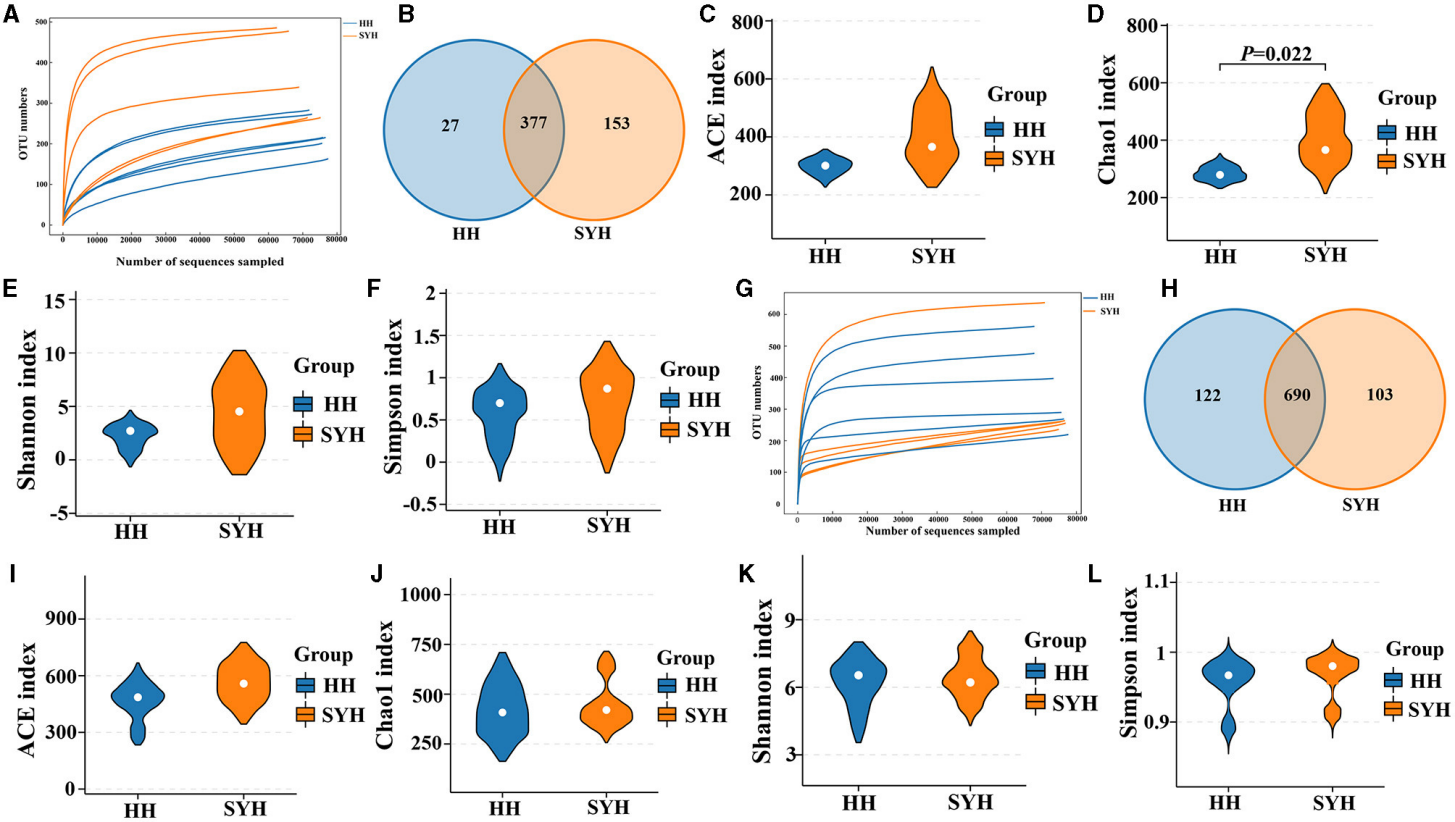
Fecal bacteria and fungi α-diversity analysis. **(A)** Bacterial rarefaction curves for the samples; **(B)** gut bacterial OTU distribution in each group; **(C)** bacterial ACE diversity; **(D)** bacterial Chao1 diversity; **(E)** bacterial Shannon diversity; **(F)** bacterial Simpson diversity; **(G)** fungal rarefaction curves for the samples; **(H)** gut fungal OTU distribution in each group; **(I)** fungal ACE diversity; **(J)** fungal Chao1 diversity; **(K)** fungal Shannon diversity; **(L)** fungal Simpson diversity. HH, common crane; SYH, demoiselle crane.

The rarefaction curves of the 16S rRNA and ITS gene sequencing analyses of the 11 fecal samples tended to flatten (Figures 1A, G), thus indicating that the microbial diversity in each sample did not increase with the increasing sequencing depth. When comparing the diversity of bacterial communities in different avian species, the results demonstrated that 377 OTUs were shared between the two crane species, which represented 93.31 and 71.13% of all the OTUs detected in common cranes (HH) and demoiselle cranes (SYH) samples, respectively (Figure 1B). It was also observed that 27 OTUs were specific to HH and 153 OTUs to SYH (Figure 1B). Regarding fungal abundance, there were 690 OTUs shared by both crane species, which represented 84.98 and 87.01% of all the OTUs detected in HH and SYH samples, respectively (Figure 1B), out of which 122 OTUs were specific to HH, and 103 OTUs to SYH (Figure 1H). Four indexes were used to analyze the bacterial and fungal α-diversity of the two cranes. The results revealed Chao1 index, which was used to measure gut bacteria α-diversity, in demoiselle cranes was remarkably higher than that in common cranes (411.07 ± 79.54 vs. 294.92 ± 22.38) (*P* < 0.05), while other indexes did not show remarkable differences (*P* > 0.05) (Figures 1C–F). There was no remarkable difference in fungal diversity (*P* > 0.05) (Figures 1I–L).

### 3.3 β-diversity analysis of the gut microbiota of the two species of cranes

**Figure 2:**
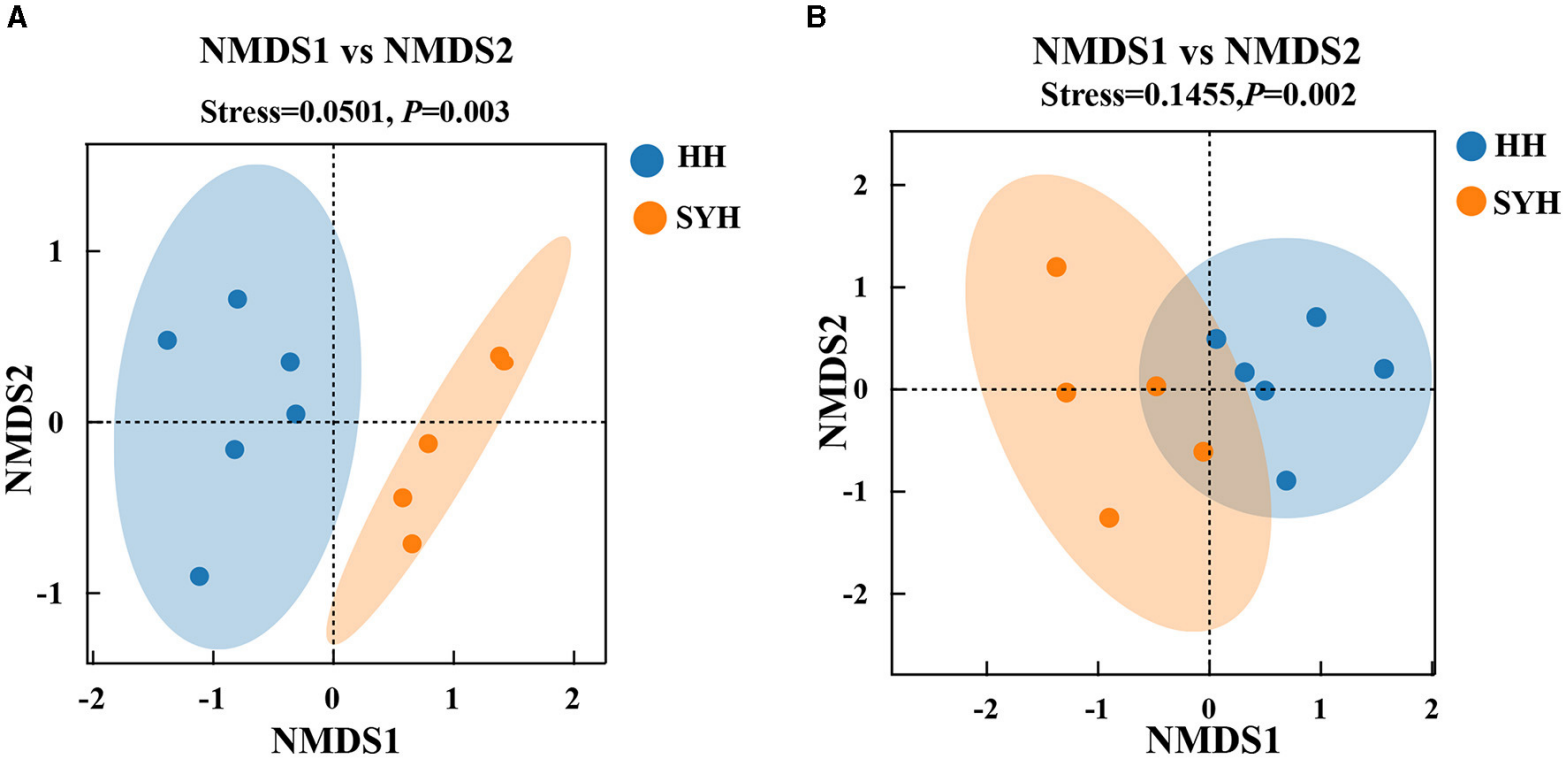
β-diversity analysis. **(A)** Unweighted UniFrac distance NMDS plots of gut bacteria; **(B)** Bray–Curtis distance NMDS plots of bacteria and fungi. HH, common crane; SYH, demoiselle crane.

The β-diversity of the bacterial communities in the different samples was analyzed using unweighted UniFrac NMDS, and the results are presented in Figure 2A. The HH and SYH samples exhibited clustering trends and a significant separation from each other. The ANOSIM test results revealed significant differences in bacterial composition among the different sample groups (*P* < 0.05). The β-diversity of the fungal communities in different samples was analyzed using Bray–Curtis NMDS, and the results are presented in Figure 2B. The HH and SYH samples exhibited clustering trends and a significant separation from each other. The ANOSIM test results demonstrated significant differences in the fungal composition among the different sample groups (*P* < 0.05).

### 3.4 Analysis of gut microbiota composition in the two species of cranes

**Figure 3:**
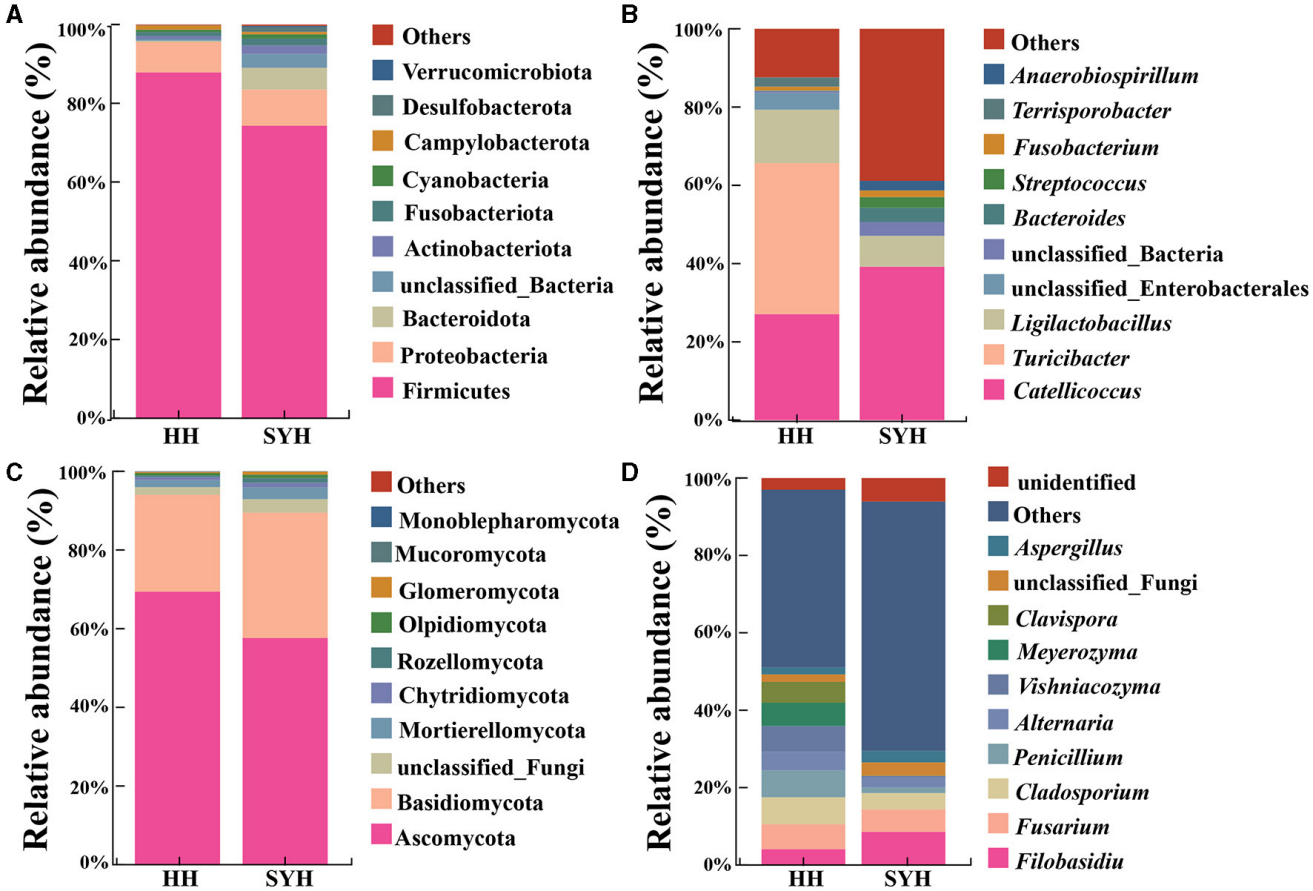
Histogram of gut bacteria and fungi composition. **(A)** The composition of the top 10 bacterial phyla according to abundance; **(B)** the top 10 bacterial genera according to abundance; **(C)** fungi with the top 10 abundances according to phylum; **(D)** the top 10 fungal genera based on abundance. HH, common crane; SYH, demoiselle crane.

An analysis of the bacterial composition revealed that, at the phylum level, the most abundant bacterial phyla in both the avian species were Firmicutes (81.06%), Proteobacteria (8.45%), Bacteroidetes (2.92%), unclassified bacteria (1.96%), Actinobacteria (1.46%), and Fusobacteriota (1.39%). Firmicutes was most abundant bacterial phylum in both HH and SYH samples (87.84 vs. 74.29%) (Figure 3A). At the genus level, the most abundant bacterial genera present in both avian species were *Catelicoccus* (33.14%), *Turicibacter* (19.32%), *Ligilactobacillus* (10.74%), unclassified *Enterobacteriales* (2.22%), unclassified_ *Bacteria* (1.96%), *Bacteroides* (1.83%), *Streptococcus* (1.42%), *Fusobacterium* (1.31%), *Terriporobacter* (1.25%), and *Anaerobiospirium* (1.19%). The most abundant bacterial genus in the HH sample was *Turicibacter* (38.60%), whereas that in the SYH sample was *Catelicoccus* (39.18%) (Figure 3B).

An analysis of fungal composition results showed that, at the phylum level, the most abundant fungal phyla present in both the avian species were Ascomycota (63.53%), Basidiomycota (28.25%), unclassified fungi (2.68%), and Mortierellomycota (2.41%). The fungal phylum exhibiting the highest abundance in both the HH and SYH samples was Ascomycota (69.42 vs. 57.63%) (Figure 3C). At the genus level, the highest abundance fungal genera were *Filobassidium* (6.34%), *Fusarium* (6.11%), *Cladosporium* (5.61%), *Penicillium* (4.20%), *Alternaria* (3.73%), *Vishniacozyma* (3.47%), *Meyerozyma* (3.02%), *Clavispora* (2.74%), unclassified fungi (2.68%), and *Aspergillus* (2.37%). *Penicillium* was the highly abundant fungal genus in the HH sample was (6.97%), while that in the SYH sample was *Filobasidium* (8.59%) (Figure 3D).

### 3.5 Analysis of differences in gut microbiota composition between the two species of cranes

**Figure 4:**
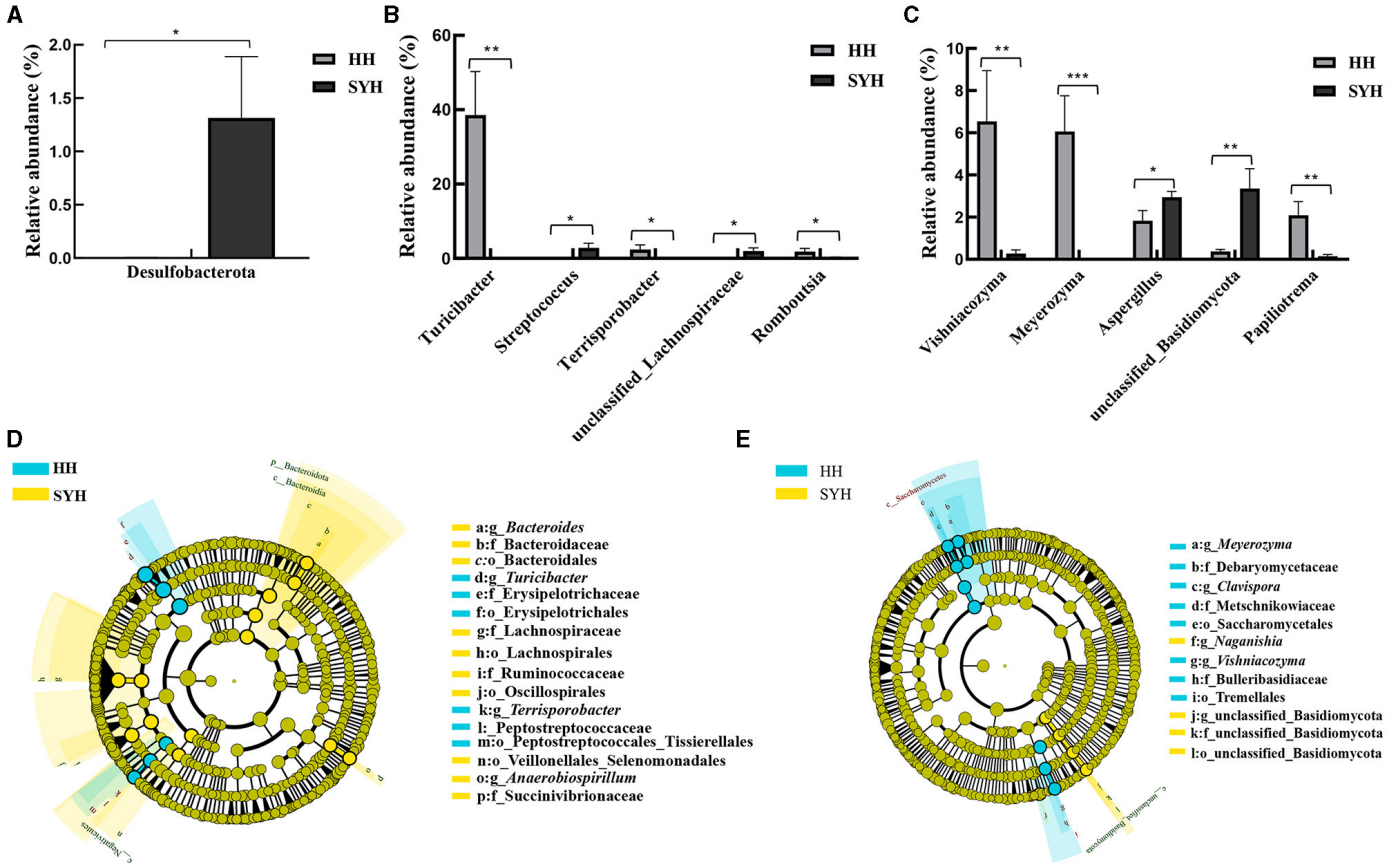
Significant differences in bacteria and fungi in the intestines of two crane species. **(A)** The bacterial phylum exhibiting significant differences based on Metastats analysis; **(B)** bacteria genera exhibiting significant differences based on Metastats analysis; **(C)** fungal genera exhibiting significant differences based on Metastats analysis; **(D)** analysis and statistics of LEfSe differences between bacterial community groups; **(E)** analysis and statistics of LEfSe differences between fungal community groups. HH, common crane; SYH, demoiselle crane.

Metastats analysis of gut bacteria and fungi with an abundance >1% and significant differences between the two crane species revealed that the bacterial phylum with significant differences between HH and SYH was Desulfobacteria (Figure 4A), which significantly abundant in SYH than in HH. Bacteria belonging to the genera *Turicibacter, Terrasporobacter*, and unclassified *Lachnospiraceae* were significantly abundant in HH samples than they were in SYH samples, while *Romboutsia* and *Streptococcus* were significantly less abundant in HH samples than they were in SYH samples (Figure 4B). Metastats analysis demonstrated that the fungal genera that were significantly higher in HH samples than they were in SYH samples were *Vishniacozyma* and *Meyerozyma*, whereas the fungal genera that were significantly lower in HH samples were *Aspergillus*, unclassified *Basidiomycota*, and *Papiliotrema* (Figure 4C).

The marker bacteria in the guts of different crane species were analyzed by LEfSe analysis. At the phylum level, HH samples did not show any marker phylum, whereas the marker phylum in SYH was *Bacteroides*. At the genus level, the marker bacterial genera of the HH sample were *Tubriciactor* and *Terriporobacter*, and those of the SYH sample were *Anaerobiospirillum* and *Bacteroides* (Figure 4D). No marker fungi were observed at the phylum level between the two crane species. At the genus level, the representative fungal genera of HH samples were *Meyerozyma, Vishniacozyma*, and *Clavispora*. The representative fungal genera in the SYH samples were unclassified *Basidiomycotta* and *Naganishia* (Figure 4E).

### 3.6 Correlation between gut bacteria and fungi

**Figure 5:**
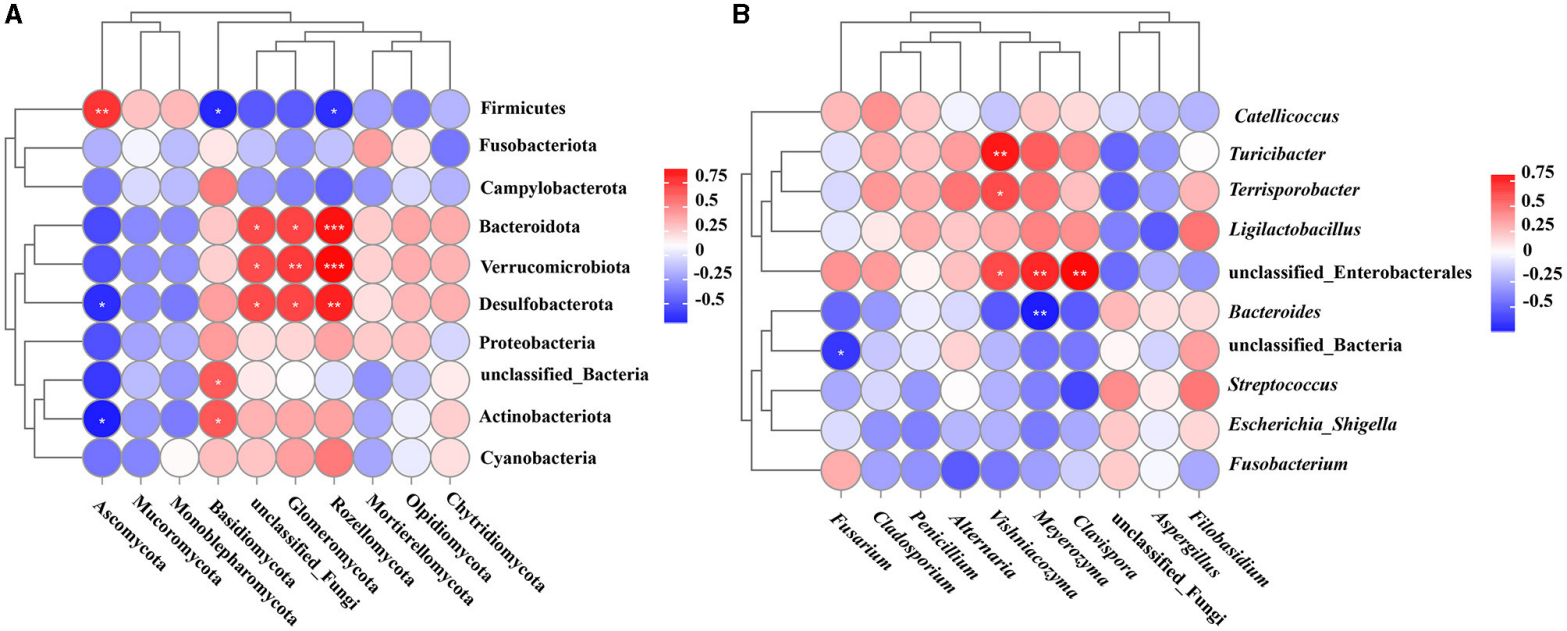
Correlation analysis of gut bacteria and fungi. **(A)** Spearman correlation analysis of bacteria and fungi with the top 10 abundance at the phylum level; **(B)** Spearman correlation analysis of bacteria and fungi with the top 10 abundance at the genus level. *P < 0.05, **P < 0.01, ***P < 0.001. HH, common crane; SYH, demoiselle crane.

Spearman correlation analysis was conducted to assess the 10 most abundant phyla of bacteria and fungi in the guts of the two crane species. The results revealed that Firmicutes exhibited a remarkable correlation with Ascomycota, Basidiomycota, and Rozellomycota (*P* < 0.01); Bacteroidota possessed a remarkable correlation with unclassified_Fungi and Glomeromycota (*P* < 0.05) and exhibited an extremely remarkable correlation with Rozellomycota (*P* < 0.001). Unclassified_Bacteria exhibited a remarkable correlation with Basidiomycota (*P* < 0.05), Actinobacteriota possessed a remarkable correlation with Ascomycota and Basidiomycota (*P* < 0.05), Desulfobacterota exhibited a remarkable correlation with Ascomycota, unclassified_Fungi, Rozellomycota and Glomeromycota (*P* < 0.01), Verrucomicrobia demonstrated a remarkable correlation with unclassified_Fungi, and Glomeromycota exhibited an extremely remarkable correlation with Rozellomycota (*P* < 0.001) (Figure 5A).

Spearman correlation analysis was performed to assess the top 10 bacterial and fungal genera in the gut of two crane species, and the results suggested that there was a remarkable correlation (*P* < 0.01) between the genera *Turicibacter* and *Vishniacozyma* (*P* < 0.01). Additionally, unclassified_Enterobacterales exhibited a remarkable correlation with *Vishniacozyma, Meyerozyma*, and *Clavispora* (*P* < 0.01), *Bacteroides* possessed a remarkable correlation with *Meyerozyma* (*P* < 0.01), and *Terrisporobacter* exhibited an extremely remarkable correlation with *Vishniacozyma* (*P* < 0.05) (Figure 5B).

## 4 Discussion

The gut microbiota plays an important role in maintaining health and life activities in animals (Zhang et al., 2021). Avian species occupy significant ecological niches. The gut microbiota of numerous rare birds has received widespread attention (Shang, 2021; Wang et al., 2021), and related research has provided important guidance for the conservation of rare species. Artificial captivity is a widely adopted measure for protecting endangered and critically endangered species (Ding and Li, 2005). There are many differences between captive and wild populations, and numerous animals released into nature cannot adapt to the wild environment, thus leading to a significant increase in their mortality rates. The most common cause of death is bacterial infection, and this may be caused by a significant increase in pathogenic groups in the gut microbiota under captive conditions and the abundance of disease-related pathways (Wasimuddin et al., 2017). Under captive conditions, animals are prone to a simple gut microbiota structure and insufficient stress response to external stimuli. Therefore, the study of gut microbiota in wild animals can provide valuable basic information for the captive protection of wild animals that are well-known hosts of new human infectious diseases (Mackenzie and Jeggo, 2013). Bird migration also facilitates the transmission of pathogens across multiple geographical ranges (Zhang et al., 2021). Therefore, the study of the gut microbiota of wild birds not only helps us to understand their life history and related mechanisms but also hinders the spread of related pathogenic microorganisms, and this is very important for the conservation and management of wild birds.

In the present study, 16S rRNA and ITS high-throughput sequencing was performed to analyze the diversity and composition of gut bacteria and fungi of common and demoiselle cranes migrating to the Yellow River Wetland. The results suggested that the α-diversity of the gut microbial of demoiselle cranes was remarkably higher than that of common cranes, and this differed from findings presented in another study analyzing the gut microbial diversity of five species of captive cranes (Wang, 2022). Studies have demonstrated that a more diverse food composition results in a higher diversity and richness of the host gut microbiota (Lau et al., 2018). Common cranes feed more on the stems, leaves, and fruits of plants. The diet of demoiselle cranes is relatively broad, and they eat both animal foods such as insects, fish, and shrimp and plant foods such as tender buds and leaves (Wang, 2022). Based on their food consumption pattern, the higher Chao1 diversity of the gut microbial of demoiselle cranes may be related to the different feeding habits of these two cranes.

At the phylum level, the bacterial phylum with the highest abundance in demoiselle and common crane samples was Firmicutes. This is consistent with the results for gut bacteria in captive common and demoiselle cranes (Wang, 2022). Research has indicated that Firmicutes are more abundant in herbivorous animals (Wang et al., 2022), and their main function is to break down cellulose into volatile fatty acids that can be assimilated by the host, increase nutrient utilization, regulate T cells to strengthen host immunity, inhibit intestinal inflammation, and maintain the ecological balance of the gut microbiota (Fernando et al., 2010; Guan et al., 2017). The abundance of Firmicutes in the guts of common cranes was higher than that in demoiselle cranes, and this may be related to the different feeding habits of the two cranes.

The diversity of the fungal microbiota in the gut is similar to that of gut bacteria and is related to factors such as the host diet, age, physical condition, and environment (Su et al., 2022). In the present study, the fungal phylum with the highest abundance in the gut of common and demoiselle cranes was Ascomycota (69.42 vs. 57.63%), and this finding was similar to the results of gut fungal studies examining wild animals such as the black-necked crane (Liu et al., 2020) and North China leopard (Hua et al., 2020). Ascomycota can promote the decomposition of plant cellulose substances (Xin et al., 2020), and the large amount of Ascomycota in the gut of common and demoiselle cranes is also related to their feeding habits and can help the host digest food and accumulate energy.

At the genus level, there were significant differences in the gut bacteria between the two crane species. *Turicibacter* was the most abundant bacterial genus in the gut of common cranes. Studies have demonstrated that *Turicibacter* is a probiotic that promotes lipid metabolism in the body (Lynch et al., 2023). Another bacterial genus with a significantly higher abundance in common crane samples than that in demoiselle cranes is *Terriporobacter*, and this bacteria is related to antioxidant capacity within the body (Li et al., 2021). The bacterial genus with the highest abundance in the gut of demoiselle cranes was *Catelicoccus*, which performs nutritional transport and bile acid hydrolysis functions (Góngora et al., 2021). The gut microbiota is greatly influenced by food. Therefore, we assumed that the food composition of the two cranes may be different and that they metabolize and accumulate energy through different gut microbiota. Additionally, the abundance of *Anaerobiospirillum* and *Bacteroides* in the gut of the demoiselle crane was significantly higher than that in the common crane. As these bacterial genera include both normal commensals and pathogens, future research will be necessary to determine the function of the species identified in cranes.

The fungal genus with the highest abundance in the gut of common cranes was *Penicillium* (6.97%), while the fungal genus with the highest abundance in the gut of demoiselle cranes was *Filobasidiella* (8.59%). *Filobasidiella* is a fungal genus that promotes anti-inflammatory activity in the body (van Thiel et al., 2022), whereas *Penicillium* belongs to the phylum Ascomycota and may be derived from the ingested food (Tang et al., 2019). These results indicate that food exerts a significant impact on the composition of gut fungi. The significantly higher abundance of fungi in the gut of the demoiselle crane was related to its anti-inflammatory ability, thus indicating that the demoiselle crane was in a state of stress due to the external environment during migration to this area. This may also be one of the reasons why the demoiselle crane continued to migrate to the wintering area after a brief stay and why the common crane overwintered in the wetlands itself.

Microorganisms residing in the gut can form symbiotic, synergistic, or antagonistic relationships that maintain the stability of the intestinal environment (Hu et al., 2016). Therefore, changes in gut bacteria and fungi can affect other bacteria and fungi by regulating the interactions between microorganisms. The correlation analysis between gut fungi and bacterial communities presented in this study indicated that there was a remarkable correlation between some bacteria and fungi with significant or insignificant changes. A previous study suggested that intestinal fungi and bacteria can interact directly with each other through physical contact or secretions, ultimately protecting the intestines from pathogen infection and maintaining homeostasis of the intestinal microbiota (Iliev and Leonardi, 2017). In the present study, a significant positive correlation was observed between *Turiciactor* and *Vishniacozyma*. *Turiciactor* is a probiotic that produces short-chain fatty acids, whereas *Vishniacozyma* is a type of yeast, so we speculated that these two beneficial microorganisms promote each other and maintain body health. The interaction between gut symbiotic fungi and bacteria predominantly occurs in the environment regulated by the mucosal immune system that plays a crucial role in maintaining the homeostasis of gut microbiota. However, the mechanism of action requires further investigation. Our study indicates that bacteria and fungi can regulate host metabolism and physiological status through mutual interactions. These results suggest that the composition of the gut microbiota varies among different birds and that the diversity of the gut microbiota is influenced by interactions between microorganisms that indirectly affect the abundance of other bacteria and fungi, thereby affecting host metabolism.

To protect common and demoiselle cranes, their gut microbiota should be regularly monitored to ascertain the health status of these cranes. Second, in the future, DNA metabarcoding technology should be utilized to analyze food composition during the resting period in the area, and relevant plants should be added in the resting and wintering areas to provide them with richer food resources.

## 5 Conclusions

The diversity and composition of gut bacteria and fungi in migrating common cranes and demoiselle cranes in the Yellow River Wetland of Baotou were analyzed using 16S rRNA and ITS high-throughput sequencing. The results revealed that Chao1 index, a measure of gut bacteria α diversity, in demoiselle cranes was remarkably higher than that in common cranes, while other indexes did not show remarkable differences. There was no remarkable difference in fungal diversity. There was a significant difference in the composition of the gut microbiota between the two crane species. At the phylum level, Firmicutes was the highly abundant bacteria in the gut of the common and demoiselle cranes, accounting for 87.84 and 74.29%, respectively. Ascomycota was the most abundant fungi in the gut of the common and demoiselle cranes. The most abundant bacterial genus in the gut of common cranes was *Turicibacter*, whereas the most abundant bacterial genus in the gut of demoiselle cranes was *Catelicoccus*. The fungal genus with the highest abundance in the gut of common cranes was *Penicillium*, whereas that with the highest abundance in the gut of demoiselle cranes was *Filobassidium*. This indicates that different crane species use different gut microbiota for food breakdown, digestion, and metabolism. The demoiselle crane sample contained certain pathogenic bacteria, thus indicating that wildlife monitoring department should focus their attention on the spread of relevant pathogenic microorganisms. Correlation analysis indicated that there was a significant correlation between gut bacteria and fungi. This study thus provides a research basis for the protection of cranes.

## Data availability statement

The original contributions presented in the study are publicly available. This data can be found here: https://www.ncbi.nlm.nih.gov/; PRJNA997114, PRJNA997117.

## Ethics statement

The animal study was approved by the Animal Ethics and Welfare Committee of Baotou Teachers College. The study was conducted in accordance with the local legislation and institutional requirements.

## Author contributions

ZL: Investigation, Methodology, Resources, Writing – original draft. TD: Formal analysis, Methodology, Writing – original draft. LW: Investigation, Resources, Writing – original draft. JW: Methodology, Visualization, Writing – original draft. YM: Investigation, Resources, Writing – original draft. DB: Methodology, Resources, Writing – original draft. LG: Project administration, Writing – original draft, Writing – review & editing. LL: Funding acquisition, Investigation, Writing – review & editing.
